# The Tyrosine Phosphatase PTPN14 Is a Negative Regulator of YAP Activity

**DOI:** 10.1371/journal.pone.0061916

**Published:** 2013-04-16

**Authors:** Chrysiis Michaloglou, Waltraut Lehmann, Typhaine Martin, Clara Delaunay, Andreas Hueber, Louise Barys, Honglin Niu, Eric Billy, Markus Wartmann, Moriko Ito, Christopher J. Wilson, Mary Ellen Digan, Andreas Bauer, Hans Voshol, Gerhard Christofori, William R. Sellers, Francesco Hofmann, Tobias Schmelzle

**Affiliations:** 1 Novartis Institutes for BioMedical Research, Disease Area Oncology, Basel, Switzerland; 2 Novartis Institutes for BioMedical Research, Developmental and Molecular Pathways, Cambridge, Massachusetts, United States of America; 3 Novartis Institutes for BioMedical Research, Center for Proteomic Chemistry, Cambridge, Massachusetts, United States of America; 4 Novartis Institutes for BioMedical Research, Developmental and Molecular Pathways, Basel, Switzerland; 5 Department of Biomedicine, Institute of Biochemistry and Genetics, University of Basel, Basel, Switzerland; 6 Novartis Institutes for BioMedical Research, Disease Area Oncology, Cambridge, Massachusetts, United States of America; Thomas Jefferson University, United States of America

## Abstract

The Hippo (Hpo) pathway is a novel signaling pathway that controls organ size in *Drosophila* and mammals and is deregulated in a variety of human cancers. It consists of a set of kinases that, through a number of phosphorylation events, inactivate YAP, a transcriptional co-activator that controls cellular proliferation and apoptosis. We have identified PTPN14 as a YAP-binding protein that negatively regulates YAP activity by controlling its localization. Mechanistically, we find that the interaction of ectopic YAP with PTPN14 can be mediated by the respective WW and PPxY motifs. However, the PTPN14 PPxY motif and phosphatase activity appear to be dispensable for the negative regulation of endogenous YAP, likely suggesting more complex mechanisms of interaction and modulation. Finally, we demonstrate that PTPN14 downregulation can phenocopy YAP activation in mammary epithelial cells and synergize with YAP to induce oncogenic transformation.

## Introduction

The Hippo (Hpo) pathway controls organ size by regulating cellular proliferation and apoptosis [1]–[3]. The pathway was originally described in *Drosophila* and comprises several kinases that are conserved in mammals. In fact, mammalian homologues of the pathway can rescue the phenotypes of corresponding *Drosophila* mutants reflecting the high degree of cross-species homology found in most Hpo pathway components [4]–[7]. Activation of the core mammalian pathway, consisting of MST-SAV and LATS-MOB complexes results in phosphorylation and inactivation of the transcriptional co-activator YAP and its family member TAZ, by means of cytoplasmic retention. Various proteins have been suggested to activate the pathway in response to contact inhibition. Members of adherens and tight junctions as well as proteins involved in cell polarity have recently been shown to regulate both YAP/TAZ activity and localisation as well as upstream components of the Hpo pathway, thus linking cell contact and polarity to cellular proliferation [8]–[10].

Consistent with its role in cellular proliferation, the Hippo pathway is implicated in cancer development [11]. Many components of the pathway have been found deregulated in human tumours and YAP itself is shown to act as an oncogene in various settings [1], [2], [12], [13]. Furthermore, the Hippo pathway has been implicated in development as well as the maintenance of stem cell populations in adult organisms [14], [15].

We have identified PTPN14 as a YAP-binding protein. PTPN14 is a non-receptor tyrosine phosphatase, which has been shown to interact with and dephosphorylate β-catenin at adherens junctions. Its potential role in cell adhesion is further supported by the fact that it contains an N-terminal FERM domain, known to link cytoplasmic proteins to the cell membrane. We report here that PTPN14 is a YAP regulator that can suppress YAP activity by promoting its cytoplasmic localisation, and provide evidence that PTPN14 downregulation can phenocopy YAP overexpression in human mammary epithelial cells and further enhance its transforming phenotype.

## Materials and Methods

### Cell culture and lentiviral transductions

293A cells and 293-FT cells were obtained from Invitrogen (Invitrogen, CA, USA). Both cell lines were maintained in DMEM supplemented with 10% (v/v) fetal calf serum (Thermo Scientific; MA, USA), 2 mM L-Glutamine, 1 mM sodium pyruvate and 0.1 mM MEM non-essential amino acids. SF268 cells were purchased from the NCI DCTD tumor/cell line repository (www.dtp.nci.nih.gov) and were maintained in RPMI-1640 supplemented with 10% (v/v) fetal calf serum, 2 mM L-Glutamine, 1 mM sodium pyruvate and 0.1 mM MEM non-essential amino acids. MCF-10A cells were obtained from ATCC (VA, USA) and maintained in DMEM/F12 medium supplemented with 5% horse serum (Invitrogen, CA, USA), 20 ng/ml EGF, 500 ng/ml hydrocortisone, 100 ng/ml Cholera toxin, 10 µg/ml insulin (all from SIGMA; MO, USA), 2 mM L-Glutamine, 1 mM sodium pyruvate and 0.1 mM MEM non-essential amino acids. Tetracycline-free FCS was used for SF268 cell lines containing doxycycline-inducible constructs (Takara Bio Europe/Clontech, France).

For lentiviral transductions, 293-FT cells were transfected with a DNA mix containing the plasmid of interest, pVPR Δ8.71 and pVSVG at a ratio of 12.5∶12.5∶1 using Lipofectamine 2000 (Invitrogen, CA) according to the manufacturer's protocol. Virus was harvested after 48 h, filtered with a 0.45-µm filter and stored at −80°C. After every viral infection, stable cell lines were established by pharmacological selection.

### QPCR-based copy number analysis

The QPCR-based copy number analysis was carried out as previously described [16]. Primers for YAP are: TGTAGTGGCACCTATCACTC and CCATCTCATCCACACTGTTC and were used at a concentration of 300 nM.

### Cloning and plasmids

YAP1 (NM_001130145; ORF length of 1515 nucleotides) was purchased from Invitrogen (Clone IOH26027; corresponds to YAP1-2γ, [17]). The YAP WW mutants were generated using the QuikChange II Site-Directed Mutagenesis Kit (Agilent Technologies, CA, USA) according to the manufacturer's protocol so that the resulting protein sequence for WW1 was mutated from W199-Q-D-P202 to A199-Q-D-A202 and the protein sequence of WW2 was mutated from W258-L-D-P261 to A258-L-D-A261. The YAP ΔPDZ mutant was generated by PCR resulting in a CDS lacking the last 241 nt of WT YAP. PTPN14 (NM_005401) was chemically synthesized by Blue Heron Biotech (WA, USA). The PTPN14 PPxY mutants were generated by mutation of the two prolines and tyrosine of each motif to alanine (PPPY to AAPA and PPEY to AAEA for the first and second PPxY motifs respectively) using QuikChange II Site-Directed Mutagenesis Kit (Agilent Technologies, CA) according to the manufacturer's protocol. The PTPN14 ΔPTP mutant was generated by PCR resulting in a PTPN14 CDS that lacked nucleotides 2725–3537.

The MCAT_Luc reporter construct was created by insertion of 10× the sequence ATTCCTC into the pGL4.27 vector (Promega; WI, USA) using the NheI and BglII sites of the MCS. The reporter was then subcloned into pLENTI6TR (Invitrogen, CA, USA) using the AgeI-ClaI sites. The pLenti6.3™-EF1α plasmid was generated by replacing the CMV promoter of pLENTI6.3/V5-DEST™ (Invitrogen, CA, USA) with the EF1α promoter. pCDNA™3.1/nV5-DEST™, pLENTI6.3/V5-DEST™ and pDEST™27 were all obtained from Invitrogen (CA, USA). Cloning of YAP constructs into pCDNA3.1/nV5-DEST™ and pLenti6.3™-EF1α and PTPN14 constructs into pDEST™27 and pLKO-TREX-HA [18] was carried out using Gateway® cloning technology (Invitrogen, CA, USA) according to the manufacturer's protocol. Cloning into pCDNA3.1/nV5 DEST™ resulted in V5-tagged YAP constructs and cloning into pDEST™27 generated GST-tagged PTPN14 constructs. The PTPN14 shRNAs were obtained from The RNAi Consortium (TRC, MISSION® TRC shRNA library, SIGMA; #5 Cat No TRCN0000006892; #6 Cat No TRCN0000367553) and were cloned into the Tet-pLKO-puro vector as previously described [19]. The GFP shRNA targets the sequence ACAACAGCCACAACGTCTATA. The shRNA sequences for YAP were previously described [20], [21] (#1 and #2 respectively). YAP siRNAs were obtained from QIAGEN (Germany) (#5 SI02662954 and #1 SI00084546).

### Antibodies

YAP1 antibody sc-101199 (Santa Cruz, CA, USA) was used for Western blot analysis, IP and immunofluorescence analysis. YAP1 antibody 2060-1 (Epitomics, CA, USA) was used for immunofluorescence analysis. PTPN14 antibody MAB4458 was obtained from R&D Systems (UK). Tubulin antibody was obtained from LabVision (MI, USA). GST antibody sc-459 and CDK4 antibody sc-601 were obtained from Santa Cruz (CA, USA) and V5 antibody R960-25 was obtained from Invitrogen (CA, USA). Secondary antibodies used for immunofluorescence were both anti-mouse and anti-rabbit Alexa Fluor® 488 and 568 (all from Invitrogen, CA, USA).

### YAP and V5 IP

For IP experiments cells were lysed in RIPA buffer (120 mM NaCl, 50 mM Tris-HCl pH 7.2, 1% Nonidet P40 (v/v), 1 mM EDTA and 0.1% (v/v) SDS), supplemented with 6 mM EGTA, 2 mM sodium orthovanadate, 6 mg/ml sodium pyrophosphate and phosSTOP and protease inhibitor cocktail (both from Roche; Switzerland). Lysates were subjected to 3 freeze-thaw cycles prior to 30 min incubation on ice for complete lysis. Lysates were then incubated with antibody for 2 h under rotation at 4°C and then incubated with 1∶5 ProtA/G bead slurry (Thermo Scientific; MA, USA) for 1 h under rotation at 4°C. Immunoprecipitates were then washed three times with RIPA buffer lacking SDS and eluted with 1× NuPAGE LDS Sample buffer (Invitrogen, CA, USA) by incubation at 95°C for 5 min.

### Protein identification by mass spectrometry

Immunoprecipitates were separated by SDS-PAGE and stained with Coomassie Blue. Protein-containing bands were excised and digested with modified porcine trypsin (Promega; WI, USA) as described [22], using a microtiter plate format (CB080, Proxeon, Odense, DK). After overnight digestion at 37 °C, peptides were eluted into a second microtiter plate with 5% formic acid and dried before analysis by LC-MS.

Peptides mixtures were separated on a 15 cm ×75 µm ProteoPep 2 PicoFrit column (New Objectives), connected to an LTQ-OrbiTrap XL mass spectrometer (Thermo). Buffer A consisted of H_2_O with 0.1% formic acid and Buffer B of 80% acetonitrile with 0.1% formic acid. Peptides were separated using a 60 min gradient from 2% B to 50% B. Data acquisition was done using a ‘Top 5 method’, where every full MS scan was followed by 5 data-dependent scans on the 5 most intense ions from the parent scan. Full scans were performed in the Orbitrap at 60'000 resolution with target values of 5 E5 ions and 500 ms injection time, while MSMS scans were done in the ion trap with 1E4 ions and 200 ms. Database searches were performed with Mascot Server using the human IPI database (version 3.64). Mass tolerances were set at 10 ppm for the full MS scans and at 0.8 Da for MSMS. Search results were validated using Scaffold v2.6 (Proteome Software) and protein identifications accepted when at least two unique peptides were detected with >95% confidence (peptide FDR 1%, protein FDR 0%).

### Luciferase assay

SF268 and 293A cells stably expressing the MCAT_Luc reporter were plated on 96-well plates in triplicate (2500 cells per well) in the presence or absence of dox. 72 h after plating medium was aspirated and cells were incubated with fresh medium containing 1.4 µM resazurin (SIGMA; MO, USA). Fluorescence measurement was made after 2 h (Ex: 540 nm, Em: 590 nm) and the cells were then lysed in fresh medium containing 1∶10 (v/v) Steady-Glo luciferase assay reagent (Promega; WI, USA) for 10 min at room temperature. Luciferase measurements were taken according to the manufacturer's protocol by transfering 75 µl of lysate to a white 96-well plate. All luciferase readings were normalized to resazurin and are depicted as the average of three independent experiments ± STDEV.

### Immunofluorescence microscopy

293A and SF268 cells were seeded onto Lab-Tek™ 8 chamber glass slides (Thermo Scientific; MA, USA) in the presence or absence of dox. Cells were fixed with 4% formaldehyde 72 h after seeding, washed twice with PBS and permeabilized with 0.5% Triton X-100/PBS solution. After washing with PBS, cells were incubated with 1.5% BSA/PBS solution (blocking solution) for 30 min at room temperature and then incubated with blocking solution containing 1∶1000 dilution of the desired antibody(ies), overnight at 4°C. Cells were then washed twice with PBS and incubated with secondary antibody in blocking buffer (1∶500) for 1 h at room temperature, protected from the light. After two washing steps with PBS, cells were incubated with 1∶1000 TO-PRO®-3 (Invitrogen; CA, USA) solution in PBS for 10 min and rinsed with water. The wells were then removed and glass slides were covered and sealed with Vectashield mounting medium (Vector laboratories; CA; USA). Microscopic analysis was performed with a confocal Leica SP5 microscope (Leica; Germany).

For quantification of nuclear and cytoplasmic YAP signal, cells were plated in clear-bottom 96-well plates and incubated in the presence or absence of dox for 72 h. Cells were fixed and stained for YAP as described above and the DNA was stained with Hoechst33342 (10 µg/ml for 10 min). Nuclear and cytoplasmic levels of YAP were quantified following image acquisition (10 fields/well) on a Cellomics automated imager (ThermoFisher; MA, USA). The average cellular Nuclear/Cytoplasmic ratio of YAP was calculated for each sample and is expressed as average of three wells ± STDEV.

### QRTPCR

For QRTPCR, RNA was collected and isolated using the RNeasy kit (QIAGEN, Germany) according to the manufacturer's protocol. Two µg of RNA for each sample were used for cDNA synthesis using the High-Capacity cDNA Reverse Transcription Kit, (Applied Biosystems; CA, USA) according to the manufacturer's protocol. QRTPCR was carried out in triplicate using the ABI PRISM® 7900HT Sequence Detector System (Applied Biosystems; CA, USA) with 5 ng of cDNA using TaqMan® probes (Applied Biosystems; CA, USA). The following TaqMan® probes were used for quantification: YAP Hs00902712_g1; PTPN14 Hs00193643_m1; β-actin Hs99999903_m1; Cyr61 HS00155479_m1; CTGF HS00170014_m1; ITGB2 HS00164957_m1; COL8A1 HS00156669_m1. Results were normalized to β-actin. Results are shown as the average of three independent experiments ± STDEV.

### Soft agar

For soft agar experiments, 3000 MCF-10A cells were plated on Costar® ultra-low attachment surface 96-well plates (Corning; NY, USA) in 100 µl medium containing agar at 0.4% final concentration. 10 µl of medium was added to the wells twice a week. Colonies were counted manually after two weeks and wells were photographed using the CloneSelect Imager (Genetix, UK).

### Anoikis assay

For induction of anoikis, 10^4^ cells were plated on Costar® ultra-low attachment surface 96-well plates (Corning; NY, USA) or tissue culture-treated 96-well plates for adherence controls, in 100 µl of medium. Anoikis was assessed after 48 hours using the cell-death detection ELISA kit (Roche Diagnostics, Germany) according to the manufacturer's protocol. Results shown are the average of three independent experiments ± STDEV.

### Clonogenic survival assay

For clonogenic survival assays, 1000 cells/well were plated in 6-well plates in duplo in the presence and absence of dox. Medium was changed twice weekly and cells were fixed and stained with crystal violet after 2–3 weeks.

## Results

**Figure 1:**
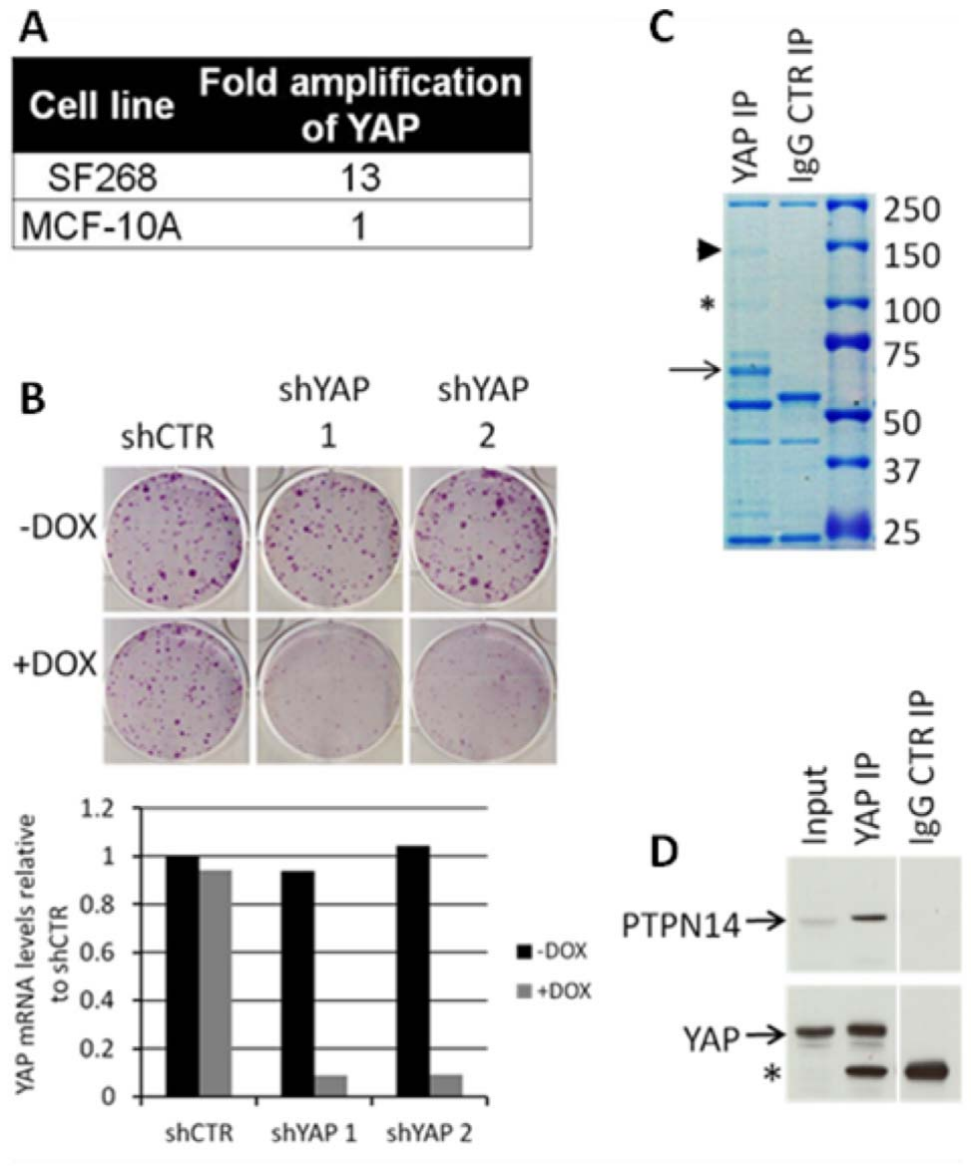
PTPN14 is a YAP-binding protein. A) QPCR-based YAP copy number analysis. MCF-10A is used as a control cell line with no YAP amplification. B) Clonogenic survival assay of SF268 cells transduced with lentivirus encoding control or two independent dox-inducible shRNAs (upper panel). QRTPCR analysis of YAP mRNA levels (lower panel). C) Coomassie-stained gel used for MS analysis of YAP-binding proteins. YAP is indicated by the black arrow, PTPN14 by the black arrowhead and the AMOT family members by an asterisk. D) IP of endogenous YAP from SF268 cell lysates. All lanes are from a single blot and exposure. Asterisk indicates the position of the IgG heavy chains.

In an attempt to identify novel regulators of YAP and the Hippo pathway we carried out a YAP immunoprecipitation (IP) experiment. As most of the current studies on YAP interaction partners have been restricted to overexpression of YAP in mechanistic settings, we decided to look at interaction partners of endogenous YAP in a cancer-relevant model. For that purpose we used the glioma cell line SF268, which expresses high levels of YAP due to genomic amplification of the gene (https://cansar.icr.ac.uk). The high levels of genomic amplification of YAP were confirmed using QPCR-based copy number analysis (Figure 1A). Furthermore, SF268 cells depend on YAP for long-term growth and survival as knock down of YAP with two independent inducible shRNAs dramatically reduced clonogenic survival of the cells (Figure 1B). On the contrary, neither hairpin affected the long-term survival of MKN45 cells (a cancer cell line harbouring a YAP deletion) in a clonogenic survival assay (data not shown), indicating that the results obtained with SF268 cells are unlikely to be an shRNA off-target artefact. Endogenous YAP was immunoprecipitated from SF268 cells and prominent bands were then excised from a Coommasie-stained gel and analyzed using mass spectrometry (MS) (Figure 1C). The MS analysis verified the presence of YAP (Figure 1C, black arrow, 31 unique peptides covering 50.8% of the sequence) as well as the presence of PTPN14 at around 140 kDa (Figure 1C black arrowhead, 42 unique peptides covering 30.2% of the sequence). AMOT family members AMOT, AMOTL1 and AMOTL2 were also prominently identified at around 90 kDa, confirming the published interaction of these proteins with YAP [23]–[27] (Figure 1C, asterisk). Importantly, the MS data were validated with a co-IP experiment of endogenous YAP and endogenous PTPN14 in SF268 cells (Figure 1D). PTPN14 has been previously identified in independent studies by MS analysis as a LATS2-binding protein in LATS2 overexpressing U2OS cells [25] and as a YAP-binding protein in FLAG-tagged YAP-overexpressing 293T and MCF-10A cells [26], [28] and HA-tagged YAP-overexpressing NIH-3T3 and MCF-10A cells [29]. Furthermore, YAP was recently identified as a PTPN14-binding protein in 293T cells [30].

**Figure 2:**
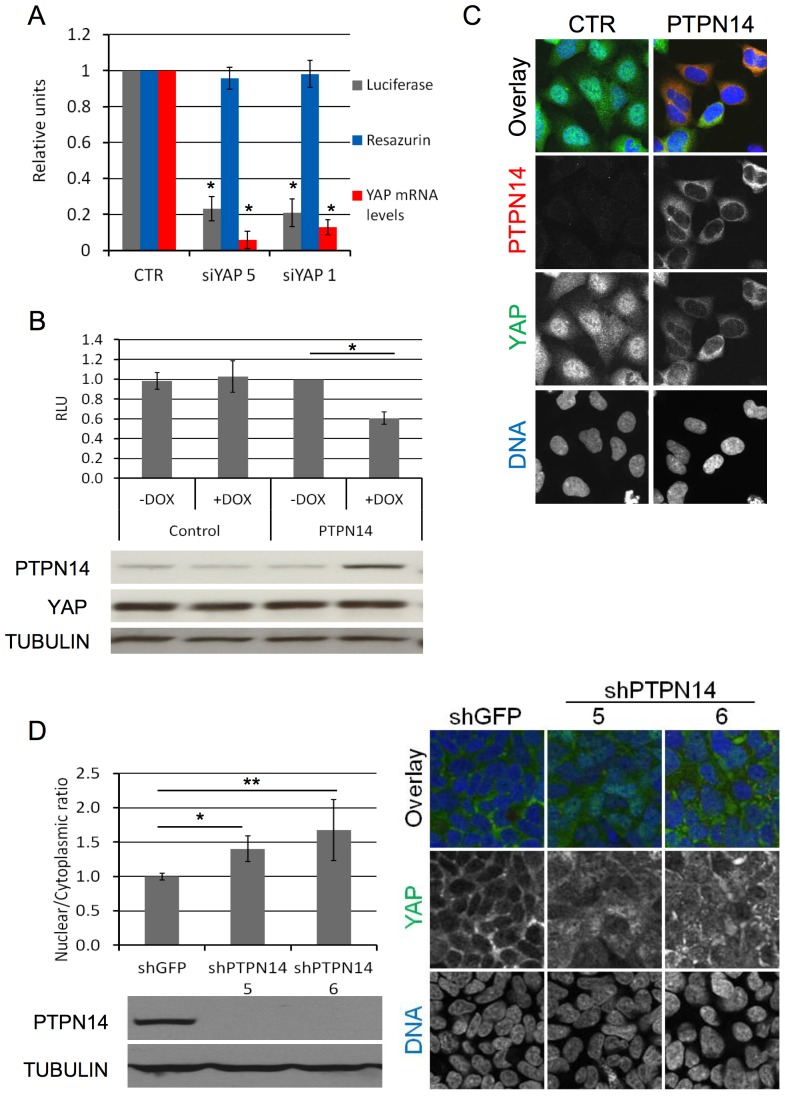
PTPN14 is a negative regulator of YAP. A) An SF268 cell line stably expressing the YAP-responsive MCAT_Luc reporter was transfected with two independent siRNAs against YAP and analysed in a Resazurin and luciferase assay 72 h after transfection. mRNA levels of YAP were determined by QRTPCR. Luciferase results are normalized based on the Resazurin results. All results are the average of 5 experiments ± STDEV. Statistical analysis was carried out with a 2-tailed paired t-test for each siRNA; * p<0.0001. B) An SF268 cell line stably expressing the YAP-responsive MCAT_Luc reporter was transduced with lentivirus encoding for dox-inducible PTPN14 expression. After pharmacological selection, luciferase expression was measured for both cell lines in the presence and absence of dox (upper panel). A Resazurin assay was carried out in parallel for each sample and used to normalize the luciferase readings. Results are shown as the average of three independent experiments ± STDEV. Statistical analysis was carried out with a 2-tailed paired t-test; * p<0.0001. RLU: relative luciferase units. PTPN14 expression achieved by dox and YAP levels in each sample was analyzed by Western blot (lower panel). Tubulin serves as loading control C) 293A cells were transduced with lentivirus encoding dox-inducible PTPN14 expression. YAP localization in control and PTPN14-expressing cell lines was analysed 72 hours post dox induction by IF at low density using confocal miroscopy. D) 293A cells were transduced with lentivirus encoding two independent dox-inducible shRNAs targeting PTPN14. The nuclear/cytoplasmic ratio of YAP was quantified at high density 72 hours post dox induction using a Cellomics automated imager with a conventional microscope, and expressed relative to the control shGFP (left upper panel). Results are shown as the average of 3 independent experiments ± STDEV. For each experiment, the average ratio was calculated from three wells per sample (10 images per well). Statistical analysis was carried out with a 2-tailed paired t-test; * p<0.001, ** p<0.05. Western blot analysis of PTPN14 levels, indicating the efficiency of each shRNA (left lower panel). Tubulin serves as a loading control. Confocal microscopy images of cells 72 hours post dox induction (right panel).

**Figure 3:**
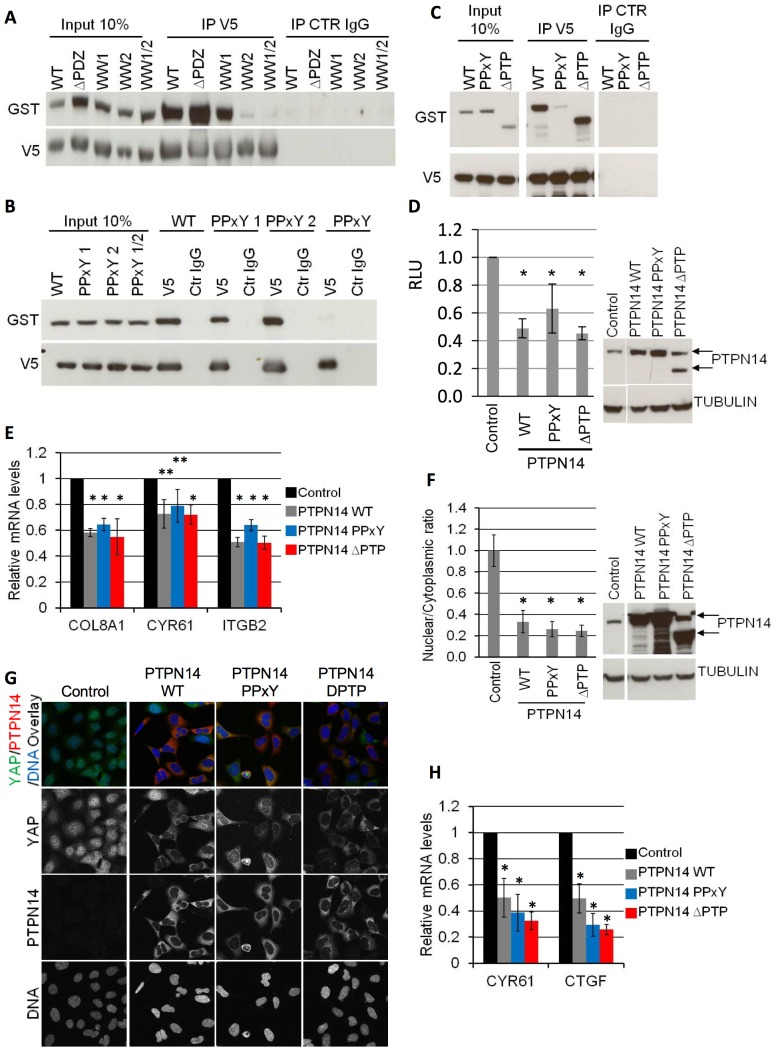
YAP-PTPN14 binding is mediated through the WW domain-PPxY motif interaction. A) 293A cells were transfected with WT GST-PTPN14 and the indicated V5-YAP constructs (WT and mutants). A V5 IP was carried out and blotted for GST to study the interaction of the various YAP mutants with WT PTPN14. Input lanes were loaded with 10% of the amount of lysate used for each IP and used to compare the expression levels of each construct. B–C) 293A cells were transfected with WT V5-YAP and the indicated GST-PTPN14 constructs (WT and mutants). A V5 IP was carried out and blotted for GST to study the interaction of the various PTPN14 mutants with WT YAP. Input lanes were loaded with 10% of the amount of lysate used for each IP and used to compare the expression levels of each construct. All lanes in (C) are from a single blot and exposure. D) An SF268 cell line stably expressing the YAP-responsive MCAT_Luc reporter was transduced with lentivirus encoding for the indicated dox-inducible PTPN14 expression. Luciferase expression of each cell line was analysed 72 hours post dox induction (left panel). A Resazurin assay was carried out in parallel for each sample and used to normalize the luciferase readings. PTPN14 expression levels achieved for each construct were analysed by Western blot (right panel; arrows indicate the WT PTPN14 protein and the truncated ΔPTP PTPN14 which migrates faster; all lanes from a single blot and exposure). Tubulin serves as loading control. Luciferase results are shown as the average of at least 3 independent experiments ± STDEV. Statistical analysis was carried out with a 2-tailed paired t-test; * p<0.05. E) The mRNA levels of the indicated YAP target genes were assessed in cells from (D) 72 hours post dox induction. Statistical analysis was carried out with a 2-tailed paired t-test; * p<0.001; **p<0.05, F) 293A cells were transduced with lentivirus encoding for the indicated dox-inducible PTPN14 expression. The nuclear/cytoplasmic YAP ratio was quantified at low density after 72 hours of dox induction using a Cellomics automated imager with a conventional microscope, and expressed relative to control (left panel). Results are shown as the average of three experiments ± STDEV. Statistical analysis was carried out with a 2-tailed paired t-test; * p<0.05. For each experiment, the average ratio was calculated from three wells per sample (10 images per well). PTPN14 expression levels were analysed by Western blot (right panel; arrows indicate the WT PTPN14 protein and the truncated ΔPTP PTPN14 which migrates faster; all lanes from a single blot and exposure). Tubulin serves as a loading control G) Confocal microscopy images of 293A cells from (F) generated 72 hours post dox induction. H) The mRNA levels of the indicated YAP target genes were assessed in cells from (F) 72 hours post dox induction. Statistical analysis was carried out with a 2-tailed paired t-test; * p<0.05.

PTPN14 is a tyrosine phosphatase, and β-catenin at adherens junctions is its only known target so far [31]. Given the link between cell adhesion and the Hippo pathway, we hypothesized that PTPN14 might not only bind to YAP but also regulate its activity in human cells. To test our hypothesis we made use of a YAP-responsive TEAD reporter in which luciferase expression is controlled by 10 consecutive MCAT sites (MCAT_Luc), a known TEAD recognition sequence [32]. The high levels of endogenous YAP in SF268 cells (Figure S1) were sufficient to drive luciferase expression from the stably integrated MCAT_Luc reporter construct, thus eliminating the need for ectopic expression of YAP (or TEAD) in this setting (Figure 2A and Figure S1). Constitutive expression of this reporter in SF268 cells was dependent on the presence of YAP, as two independent YAP siRNAs reduced luciferase expression by approximately 80%, notably in absence of significant short-term impact on cell viability as assessed by Resazurin (Figure 2A). Overexpression of PTPN14 in SF268 cells stably expressing the MCAT_Luc reporter reduced luciferase activity by approximately 40% (Figure 2B). This was accompanied by a decrease in the mRNA levels of YAP target genes *COL8A1*, *CYR61 and ITGB2* (Figure 3E). In agreement with this, overexpression of PTPN14 in 293A cells [33], [34] resulted in nuclear exclusion of YAP at low density (Figure 2C) and the downregulation of YAP target genes *CYR61* and *CTGF* (Figure 3H). Furthermore, knockdown of PTPN14 by two independent shRNA constructs in 293A cells resulted in approximately 1.5-fold increase of nuclear YAP at high density (Figure 2D). Finally, downregulation of PTPN14 expression levels in SF268 cells resulted in increased levels of nuclear YAP at high density (Figure S2). We therefore conclude that PTPN14 is a negative regulator of YAP, and it can reduce YAP transcriptional activity by inducing YAP nuclear exclusion and/or promoting YAP cytoplasmic retention.

We then aimed at identifying the domains of YAP and PTPN14 that are responsible for the interaction of the two proteins as well as for the regulation of YAP by PTPN14. GST-tagged wild type (WT) PTPN14 and V5-tagged YAP mutants were co-expressed transiently in 293A cells and the ability of the YAP mutants to bind to PTPN14 was tested by a V5 co-IP (Figure 3A). Deletion of the PDZ domain (ΔPDZ) or mutation of the first WW domain of YAP (WW1) had no effect on the ability of PTPN14 to co-IP with YAP. However, mutation of the second WW domain of YAP (WW2) significantly reduced the binding of the two proteins, indicating that the second WW domain of YAP is necessary for binding to PTPN14. Similarly, co-expression of GST-tagged PTPN14 mutants and V5-tagged WT YAP indicated that mutation of both PPxY motifs of PTPN14 (PPxY) was necessary to abrogate YAP-PTPN14 binding (Figure 3B). Deletion of the phosphatase domain (ΔPTP) had no effect on PTPN14-YAP binding (Figure 3C). We therefore conclude that, at least in the mechanistic setting of ectopic overexpression, the YAP-PTPN14 interaction is mediated by the second WW domain of YAP and either of the PPxY motifs of PTPN14. The interaction of WW domain of YAP with the PPxY motif of PTPN14 is not unexpected as the binding of the WW domain to the PPxY motif has been identified for a variety of protein interactions [35], [36]. In fact, the WW-PPxY interaction is utilized by a variety of binding proteins that associate with YAP [37].

Furthermore, we tested the ability of various PTPN14 mutants to regulate endogenous YAP activity and localization. Interestingly, expression of the PPxY mutant or the ΔPTP mutant downregulated luciferase expression in SF268 MCAT_Luc cells to levels comparable to WT PTPN14 (Figure 3D). In parallel, YAP target genes *COL8A1*, *CYR61 and ITGB2* were also downregulated similarly to PTPN14 WT expression (Figure 3E). Of note, expression of an unrelated protein had no effect on luciferase levels and YAP target gene mRNA levels (data not shown), indicating that the PTPN14 findings are not an artefact of ectopic expression in these cells. In agreement with these results, ectopic expression of the PPxY or ΔPTP PTPN14 mutants in 293A cells still resulted in the nuclear exclusion of endogenous YAP at low density (Figure 3F and G) and downregulation of YAP target genes *CYR61* and *CTGF* (Figure 3H). We therefore conclude that, at least in the physiological setting with endogenous (rather than ectopic) YAP (over)expression, the PPxY mutant of PTPN14 does not appear to be sufficient to abrogate negative regulation of YAP, and neither is the loss of PTPN14 phosphatase activity.

**Figure 4:**
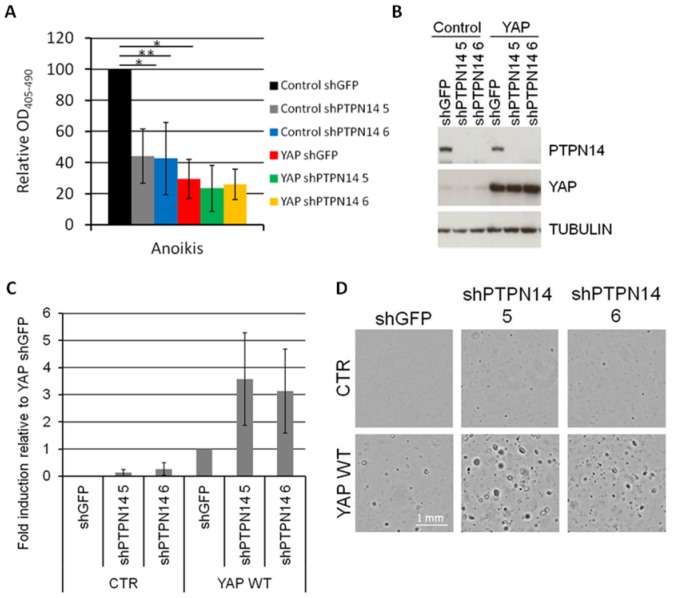
Down-regulation of PTPN14 enhances the oncogenic phenotype of YAP. A) MCF-10A cells were transduced with lentivirus encoding for YAP expression and after pharmacological selection transduced with lentivirus encoding for dox-inducible shRNAs targeting either PTPN14 or GFP (control) (two independent shRNAs targeting PTPN14). All six cell lines were subjected to an anoikis assay in low-attachment plates in the presence of dox for 48 hours. Results are shown as the average of three experiments ± STDEV. Statistical analysis was carried out with a 2-tailed paired t-test; * p<0.05, ** p = 0.05. B) Western blot analysis of cells from (A) 48 hours post dox induction. Tubulin serves as a loading control. C) Soft agar assay quantification of cells from (A). Results were obtained two weeks post dox induction and are the average of three experiments ± STDEV. D) Images of wells from (C) at the time of quantification. Scale: 1 mm.

YAP has been shown to act as an oncogene in MCF-10A cells by protecting from anoikis and inducing EMT and transformation [12]. Interestingly, knockdown of PTPN14 also protected cells from anoikis-mediated cell death to an extent similar to that achieved by YAP overexpression (Figure 4A–B). Knockdown of PTPN14 in YAP-overexpressing cells did not have a significant effect on anoikis-mediated cell death levels. Although this could possibly be due to limitations of the anoikis assay it could also suggest that the effect of PTPN14 in anoikis-mediated cells death of MCF-10A cells is YAP-dependent. Finally, we tested the ability of the PTPN14 hairpins to induce transformation of MCF-10A cells. In the control MCF-10A cell line, knockdown of PTPN14 caused an increase in the number of colonies growing in soft agar, on average 20% of the number achieved by YAP overexpression (Figure 4C–D). However, in the presence of ectopic YAP MCF-10A cells with PTPN14 knockdown formed approximately 3 times more colonies than cells with YAP overexpression alone, indicating that downregulation of PTPN14 can further promote the transforming phenotype of YAP (Figure 4C–D). We conclude that PTPN14 downregulation can phenocopy YAP activation with respect to protection from anoikis and possibly transformation in human cells and it can further promote YAP-mediated transformation.

## Discussion

We have demonstrated that PTPN14 is a YAP-binding protein that can inactivate YAP by promoting nuclear exclusion and/or cytoplasmic retention at low density in human cells, resulting in downregulation of YAP target genes. Mechanistically, the interaction between YAP and PTPN14 can be mediated through the WW2 domain of YAP and the PPxY motifs of PTPN14. The nature of the YAP-PTPN14 interaction has also been studied in three previous reports, which also indicate that the YAP-PTPN14 interaction is mediated by WW-PPxY binding [28]–[30]. However, there is a discrepancy with regards to the contribution of the individual domains to the binding between all four studies. Liu *et al.* demonstrate that loss of either WW domain can abrogate binding whereas Huang *et al.* show that loss of both domains is necessary to disrupt binding, Wang et al identify the second WW domain being more important than the first for this interaction and we show that the second WW domain is primarily responsible for YAP-PTPN14 binding. With regard to the PPxY motifs, Liu *et al.* and we demonstrate that loss of both is necessary for abrogation of YAP-binding whereas Huang *et al.* and Wang et al. indicate that deletion of either motif dramatically reduces the interaction of the two proteins. The reason for these differences is not yet clear. The discrepancy might in part be due to the different methods used to create the mutants in each study: for the PPxY motif, mutation of tyrosine residue alone [28], [29] or mutation of both prolines and the tyrosine to alanine (this study) vs full deletion of the entire motif [30]; and for the WW domains, mutation of the tryptophan and proline residues to alanine (this study) vs full deletion of the domain [28]–[30]. Most importantly however, all four studies indicate that the YAP-PTPN14 interaction is mediated by WW domain-PPxY motif binding.

We find that loss of YAP-binding through mutation of the PPxY motifs, as determined by biochemical IP in an ectopic expression setting, does not appear to abrogate regulation of endogenous YAP activity and localization by PTPN14. Opposite to our findings, Liu *et al.* and Huang *et al.* show that mutation of both PPxY motifs of PTPN14 can, albeit only partially, abrogate negative regulation of YAP activity using a reporter gene assay (RGA). Notably, these studies rely on the ectopic overexpression of both PTPN14 and YAP (i.e., identical to the mechanistic IP setting), to assess the impact of binding mutants in RGA, whereas we explore the functional PTPN14 domain contributions on the activity of endogenous YAP in models which possibly implicate physiological formation of larger complexes (see below). However, and again opposite to our findings, Wang *et al*. demonstrate that deletion of both PPxY motifs abolishes PTPN14-mediated regulation of endogenous YAP localisation in MCF-10A cells. The difference between our data and of the report of Wang *et al*. could, at least in part, be caused by the fact that we used alanine substitutions to generate the PTPN14 mutants whereas Wang *et al*. deleted the entire motif, which could result in a stronger phenotype. Finally, the discrepancies between the four studies could potentially reflect model or cell-line specific differences in PTPN14-mediated regulation of YAP.

Our results indicate that mutation of the two PPxY motifs of PTPN14 can disrupt YAP-binding but cannot abolish PTPN14-mediated regulation of YAP. Importantly, it should be noted that apart from two PPxY motifs, PTPN14 contains an LPxY and a VPxY motif, both of which have been shown to interact with WW domains *in vitro*, albeit with lower affinity than the PPxY motif [38], [39]. It is therefore possible that binding of the WW domains of endogenous YAP to the LPxY and VPxY motifs of (PPxY-mutated), overexpressed PTPN14 would be sufficient to mediate the translocation of endogenous YAP to the cytoplasm as shown in figure 3G and to downregulate endogenous YAP activity in an RGA as shown in figure 3D. Furthermore, it is possible that the interaction between ectopically-expressed WT YAP and PPxY-mutant PTPN14 would not be detectable in an IP experiment as shown in figure 3B, due to the reduced affinity of the remaining LPxY and VPxY motifs for the WW domains of YAP. Further mutating the LPxY and VPxY motifs in PTPN14 should bypass the problem of motif redundancy within the protein and provide further insight as to the role of YAP-binding in the regulation of its activity and localization.

Another possible explanation for the uncoupling of YAP-binding and YAP regulation by PTPN14 as indicated by our experiments is that PTPN14 and YAP are part of a larger complex, possibly formed at adherens junctions, and in line with the findings for the Pez/Kibra interaction in *Drosophila* (30), with the complex components interacting through multiple domains. Although loss of the PPxY motif abolishes binding to YAP as demonstrated by mechanistic co-IP experiments, it is conceivable that this does not destabilize a potentially larger complex sufficiently to completely abrogate PTPN14-mediated regulation of YAP localization and activity in a physiological setting. Yet another possibility is that PTPN14 can function as a dimer/multimer. In the presence of the ectopic PPxY mutant, the endogenous wild-type protein could heterodimerize with the ectopic mutant and create dimers that, although not interacting strongly enough with YAP to detect by co-IP experiments, can still regulate YAP localization and activity in a physiological setting.

We demonstrate that the phosphatase activity of PTPN14 is dispensable for YAP regulation. This is shown not only in an RGA, but also by means of expression levels of YAP target genes which are similar to those seen by expression of WT PTPN14, as well as YAP localization which remains cytoplasmic at low density in the presence of the ΔPTP mutant. Furthermore, and in agreement with our findings, the PTP domain is also dispensable for PTPN14-mediated control of YAP localization in MCF-10A cells as well as for the growth-regulatory role of Pez in the *Drosophila* midgut and wing development [30], [40]. However, in their recent publication, Liu and colleagues claim that YAP is a direct target of PTPN14. Although we cannot formally exclude that YAP is targeted by PTPN14 under certain circumstances or in certain cell types, we fail to see a role for the PTP domain in YAP-binding and regulation in our setting. As the Y357 phosphorylation which according to Liu *et al*. is targeted by PTPN14, has been implicated in the regulation of YAP's tumor suppressor phenotype in response to DNA damage [21], it is possible that tyrosine dephosphorylation of YAP by PTPN14 is more relevant in that setting.

Tyrosine phosphatases are known to stabilize adherens junctions by means of counteracting tyrosine kinases. However, deletion of the phosphatase domain does not affect the ability of PTPN14 to regulate YAP activity and localization, indicating that PTPN14 might have both phosphatase-dependent functions at adherens junctions and phosphatase-independent functions in regulating YAP activity. Whether PTPN14-mediated YAP regulation is at all linked to the presence of functional adherens junctions remains to be elucidated.

Lastly, we provide evidence that PTPN14 downregulation can phenocopy YAP over-expression in its ability to rescue MCF-10A cells from anoikis. Furthermore, and in agreement with Wang *et al*. [30], we show that downregulation of PTPN14 can induce transformation of MCF-10A cells and can exacerbate the transforming phenotype of ectopic YAP in MCF-10A cells. While the induction of transformation upon PTPN14 downregulation - which results in prolonged nuclear localization/activity of YAP ((Figure 2D and Figure S2) and other reports [28], [30]) - may be attributed to a number of downstream events, a key contribution is likely provided by the increased survival under loss of matrix attachment, as evidenced by the anoikis data (Figure 4A). Together with the finding that PTPN14 loss can induce aberrant acini formation in MCF-10A cells [30], these results indicate a potential tumor suppressive role for PTPN14 in human cells, in agreement with previous reports of PTPN14 loss of heterozygosity (LOH) in colorectal cancer [41].

## Supporting Information

Figure S1 **Comparison of luciferase reporter activity in SF268 and 293A cells.** SF268 and 293A cells were transduced with lentivirus encoding for the YAP/TEAD-responsive MCAT luciferase reporter. Luciferase expression in each stable cell line was analysed in parallel in the absence of exogenous YAP (left panel). The same number of cells was plated for each cell line and a luciferase and resazurin assay was carried out 72 hours after plating. Luciferase expression was normalized based on Resazurin readings. WB analysis of YAP levels in SF268 and 293A cells (right panel). CDK4 serves as loading control. s.e.: short exposure; l.e.: long exposure. All lanes are from a single blot and exposure. The lane after SF268 was left empty.
(TIF)

Figure S2 **YAP localization in SF268 cells.** SF268 cells were transduced with lentivirus encoding for constitutive PTPN14 knock down. Stable cell lines were plated onto Lab-Tek™ 8 chamber glass slides and allowed to reach confluency. Seventy-two hours after seeding, cell lines were analysed by immunofluorescence for PTPN14 and YAP expression using a confocal microscope. DAPI was used for DNA staining.
(TIF)

## References

[1] Camargo FD, Gokhale S, Johnnidis JB, Fu D, Bell GW, et al (2007) YAP1 increases organ size and expands undifferentiated progenitor cells. Curr Biol 17: 2054–2060. 17980593 10.1016/j.cub.2007.10.039

[2] Dong J, Feldmann G, Huang J, Wu S, Zhang N, et al (2007) Elucidation of a universal size-control mechanism in Drosophila and mammals. Cell 130: 1120–1133. 17889654 10.1016/j.cell.2007.07.019 PMC2666353

[3] Zhao B, Lei QY, Guan KL (2008) The Hippo-YAP pathway: new connections between regulation of organ size and cancer. Curr Opin Cell Biol 20: 638–646. 18955139 10.1016/j.ceb.2008.10.001 PMC3296452

[4] Huang J, Wu S, Barrera J, Matthews K, Pan D (2005) The Hippo signaling pathway coordinately regulates cell proliferation and apoptosis by inactivating Yorkie, the Drosophila Homolog of YAP. Cell 122: 421–434. 16096061 10.1016/j.cell.2005.06.007

[5] Lai ZC, Wei X, Shimizu T, Ramos E, Rohrbaugh M, et al (2005) Control of cell proliferation and apoptosis by mob as tumor suppressor, mats. Cell 120: 675–685. 15766530 10.1016/j.cell.2004.12.036

[6] Tao W, Zhang S, Turenchalk GS, Stewart RA, St John MA, et al (1999) Human homologue of the Drosophila melanogaster lats tumour suppressor modulates CDC2 activity. Nat Genet 21: 177–181. 9988268 10.1038/5960

[7] Wu S, Huang J, Dong J, Pan D (2003) hippo encodes a Ste-20 family protein kinase that restricts cell proliferation and promotes apoptosis in conjunction with salvador and warts. Cell 114: 445–456. 12941273 10.1016/s0092-8674(03)00549-x

[8] Boggiano JC, Fehon RG (2012) Growth control by committee: intercellular junctions, cell polarity, and the cytoskeleton regulate Hippo signaling. Dev Cell 22: 695–702. 22516196 10.1016/j.devcel.2012.03.013 PMC3376383

[9] Genevet A, Tapon N (2011) The Hippo pathway and apico-basal cell polarity. Biochem J 436: 213–224. 21568941 10.1042/BJ20110217

[10] Zhao B, Tumaneng K, Guan KL (2011) The Hippo pathway in organ size control, tissue regeneration and stem cell self-renewal. Nat Cell Biol 13: 877–883. 21808241 10.1038/ncb2303 PMC3987945

[11] Pan D (2010) The hippo signaling pathway in development and cancer. Dev Cell 19: 491–505. 20951342 10.1016/j.devcel.2010.09.011 PMC3124840

[12] Overholtzer M, Zhang J, Smolen GA, Muir B, Li W, et al (2006) Transforming properties of YAP, a candidate oncogene on the chromosome 11q22 amplicon. Proc Natl Acad Sci U S A 103: 12405–12410. 16894141 10.1073/pnas.0605579103 PMC1533802

[13] Schlegelmilch K, Mohseni M, Kirak O, Pruszak J, Rodriguez JR, et al (2011) Yap1 acts downstream of alpha-catenin to control epidermal proliferation. Cell 144: 782–795. 21376238 10.1016/j.cell.2011.02.031 PMC3237196

[14] Mauviel A, Nallet-Staub F, Varelas X (2012) Integrating developmental signals: a Hippo in the (path)way. Oncogene 31: 1743–1756. 21874053 10.1038/onc.2011.363

[15] Varelas X, Wrana JL (2012) Coordinating developmental signaling: novel roles for the Hippo pathway. Trends Cell Biol 22: 88–96. 22153608 10.1016/j.tcb.2011.10.002

[16] Ito M, Barys L, O'Reilly T, Young S, Gorbatcheva B, et al (2011) Comprehensive mapping of p53 pathway alterations reveals an apparent role for both SNP309 and MDM2 amplification in sarcomagenesis. Clin Cancer Res 17: 416–426. 21159888 10.1158/1078-0432.CCR-10-2050

[17] Gaffney CJ, Oka T, Mazack V, Hilman D, Gat U, et al (2012) Identification, basic characterization and evolutionary analysis of differentially spliced mRNA isoforms of human YAP1 gene. Gene 509: 215–222. 22939869 10.1016/j.gene.2012.08.025 PMC3455135

[18] Wee S, Wiederschain D, Maira SM, Loo A, Miller C, et al (2008) PTEN-deficient cancers depend on PIK3CB. Proc Natl Acad Sci U S A 105: 13057–13062. 18755892 10.1073/pnas.0802655105 PMC2529105

[19] Wiederschain D, Wee S, Chen L, Loo A, Yang G, et al (2009) Single-vector inducible lentiviral RNAi system for oncology target validation. Cell Cycle 8: 498–504. 19177017 10.4161/cc.8.3.7701

[20] Basu S, Totty NF, Irwin MS, Sudol M, Downward J (2003) Akt phosphorylates the Yes-associated protein, YAP, to induce interaction with 14-3-3 and attenuation of p73-mediated apoptosis. Mol Cell 11: 11–23. 12535517 10.1016/s1097-2765(02)00776-1

[21] Levy D, Adamovich Y, Reuven N, Shaul Y (2008) Yap1 phosphorylation by c-Abl is a critical step in selective activation of proapoptotic genes in response to DNA damage. Mol Cell 29: 350–361. 18280240 10.1016/j.molcel.2007.12.022

[22] Shevchenko A, Wilm M, Vorm O, Mann M (1996) Mass spectrometric sequencing of proteins silver-stained polyacrylamide gels. Anal Chem 68: 850–858. 8779443 10.1021/ac950914h

[23] Chan SW, Lim CJ, Chong YF, Pobbati AV, Huang C, et al (2011) Hippo pathway-independent restriction of TAZ and YAP by angiomotin. J Biol Chem 286: 7018–7026. 21224387 10.1074/jbc.C110.212621 PMC3044958

[24] Oka T, Schmitt AP, Sudol M (2012) Opposing roles of angiomotin-like-1 and zona occludens-2 on pro-apoptotic function of YAP. Oncogene 31: 128–134. 21685940 10.1038/onc.2011.216

[25] Paramasivam M, Sarkeshik A, Yates JR 3rd, Fernandes MJ, McCollum D (2011) Angiomotin family proteins are novel activators of the LATS2 kinase tumor suppressor. Mol Biol Cell 22: 3725–3733. 21832154 10.1091/mbc.E11-04-0300 PMC3183025

[26] Wang W, Huang J, Chen J (2011) Angiomotin-like proteins associate with and negatively regulate YAP1. J Biol Chem 286: 4364–4370. 21187284 10.1074/jbc.C110.205401 PMC3039387

[27] Zhao B, Li L, Lu Q, Wang LH, Liu CY, et al (2011) Angiomotin is a novel Hippo pathway component that inhibits YAP oncoprotein. Genes Dev 25: 51–63. 21205866 10.1101/gad.2000111 PMC3012936

[28] Liu X, Yang N, Figel SA, Wilson KE, Morrison CD, et al.. (2012) PTPN14 interacts with and negatively regulates the oncogenic function of YAP. Oncogene. 10.1038/onc.2012.147 PMC4402938 22525271

[29] Huang JM, Nagatomo I, Suzuki E, Mizuno T, Kumagai T, et al.. (2012) YAP modifies cancer cell sensitivity to EGFR and survivin inhibitors and is negatively regulated by the non-receptor type protein tyrosine phosphatase 14. Oncogene. 10.1038/onc.2012.231 PMC3443515 22689061

[30] Wang W, Huang J, Wang X, Yuan J, Li X, et al (2012) PTPN14 is required for the density-dependent control of YAP1. Genes Dev 26: 1959–1971. 22948661 10.1101/gad.192955.112 PMC3435498

[31] Wadham C, Gamble JR, Vadas MA, Khew-Goodall Y (2003) The protein tyrosine phosphatase Pez is a major phosphatase of adherens junctions and dephosphorylates beta-catenin. Mol Biol Cell 14: 2520–2529. 12808048 10.1091/mbc.E02-09-0577 PMC194899

[32] Jiang SW, Desai D, Khan S, Eberhardt NL (2000) Cooperative binding of TEF-1 to repeated GGAATG-related consensus elements with restricted spatial separation and orientation. DNA Cell Biol 19: 507–514. 10975468 10.1089/10445490050128430

[33] Miller E, Yang J, DeRan M, Wu C, Su AI, et al (2012) Identification of serum-derived sphingosine-1-phosphate as a small molecule regulator of YAP. Chem Biol 19: 955–962. 22884261 10.1016/j.chembiol.2012.07.005

[34] Yu FX, Zhao B, Panupinthu N, Jewell JL, Lian I, et al (2012) Regulation of the Hippo-YAP pathway by G-protein-coupled receptor signaling. Cell 150: 780–791. 22863277 10.1016/j.cell.2012.06.037 PMC3433174

[35] Bork P, Sudol M (1994) The WW domain: a signalling site in dystrophin? Trends Biochem Sci 19: 531–533. 7846762 10.1016/0968-0004(94)90053-1

[36] Chen HI, Sudol M (1995) The WW domain of Yes-associated protein binds a proline-rich ligand that differs from the consensus established for Src homology 3-binding modules. Proc Natl Acad Sci U S A 92: 7819–7823. 7644498 10.1073/pnas.92.17.7819 PMC41237

[37] Webb C, Upadhyay A, Giuntini F, Eggleston I, Furutani-Seiki M, et al (2011) Structural features and ligand binding properties of tandem WW domains from YAP and TAZ, nuclear effectors of the Hippo pathway. Biochemistry 50: 3300–3309. 21417403 10.1021/bi2001888

[38] Chen HI, Einbond A, Kwak SJ, Linn H, Koepf E, et al (1997) Characterization of the WW domain of human yes-associated protein and its polyproline-containing ligands. J Biol Chem 272: 17070–17077. 9202023 10.1074/jbc.272.27.17070

[39] Fotia AB, Ekberg J, Adams DJ, Cook DI, Poronnik P, et al (2004) Regulation of neuronal voltage-gated sodium channels by the ubiquitin-protein ligases Nedd4 and Nedd4-2. J Biol Chem 279: 28930–28935. 15123669 10.1074/jbc.M402820200

[40] Poernbacher I, Baumgartner R, Marada SK, Edwards K, Stocker H (2012) Drosophila Pez acts in Hippo signaling to restrict intestinal stem cell proliferation. Curr Biol 22: 389–396. 22305752 10.1016/j.cub.2012.01.019

[41] Wang Z, Shen D, Parsons DW, Bardelli A, Sager J, et al (2004) Mutational analysis of the tyrosine phosphatome in colorectal cancers. Science 304: 1164–1166. 15155950 10.1126/science.1096096

