# Supplementary figures and images for: The Tyrosine Phosphatase PTPN14 Is a Negative Regulator of YAP Activity
Source: PLoS One. 2013 Apr 16;8(4):e61916. doi: 10.1371/journal.pone.0061916 (PMC3628344; doi:10.1371/journal.pone.0061916)

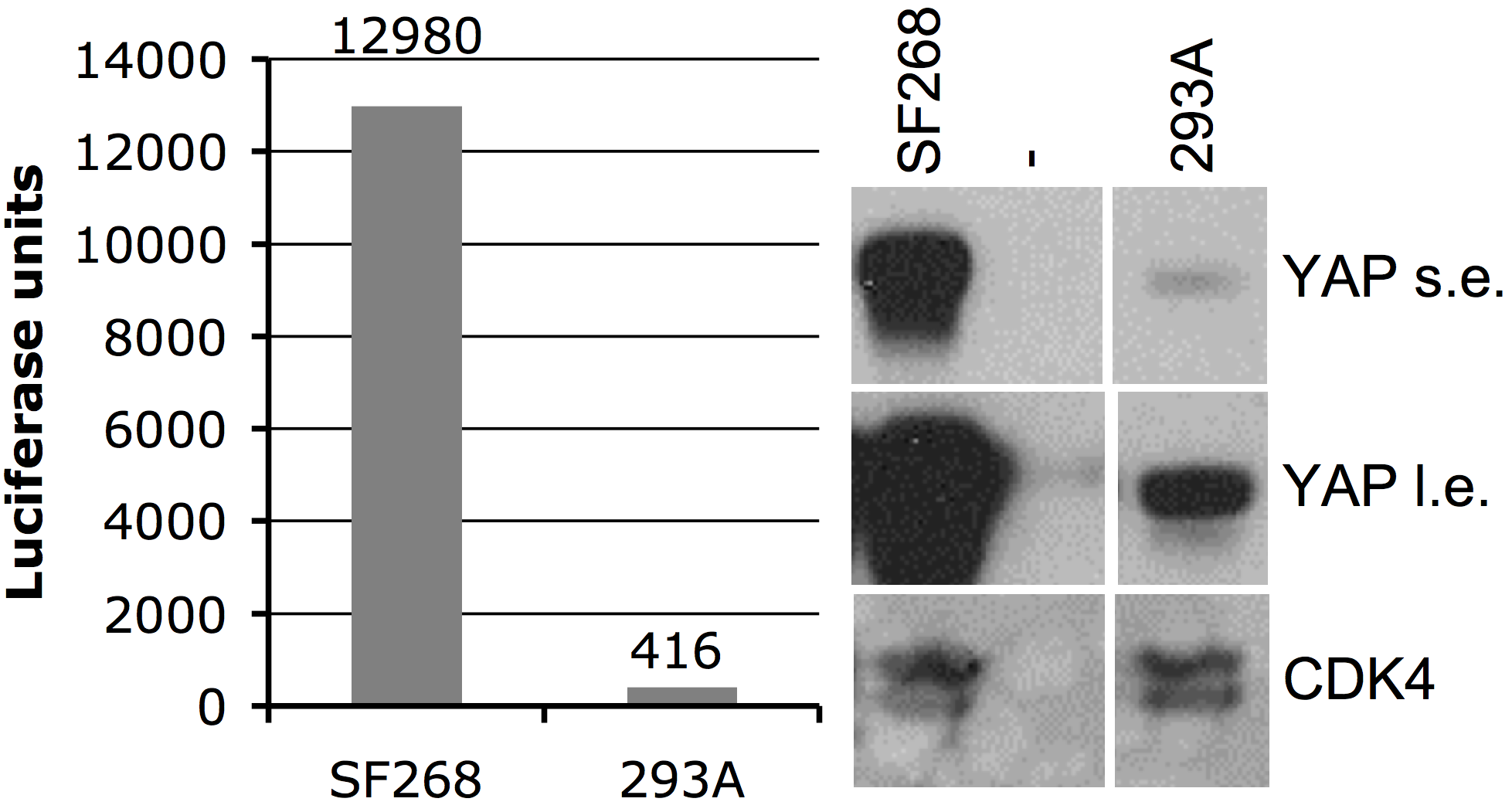

Supplement: Figure S1 — Comparison of luciferase reporter activity in SF268 and 293A cells. SF268 and 293A cells were transduced with lentivirus encoding for the YAP/TEAD-responsive MCAT luciferase reporter. Luciferase expression in each stable cell line was analysed in parallel in the absence of exogenous YAP (left panel). The same number of cells was plated for each cell line and a luciferase and resazurin assay was carried out 72 hours after plating. Luciferase expression was normalized based on Resazurin readings. WB analysis of YAP levels in SF268 and 293A cells (right panel). CDK4 serves as loading control. s.e.: short exposure; l.e.: long exposure. All lanes are from a single blot and exposure. The lane after SF268 was left empty. (TIF) [file pone.0061916.s001.tif]

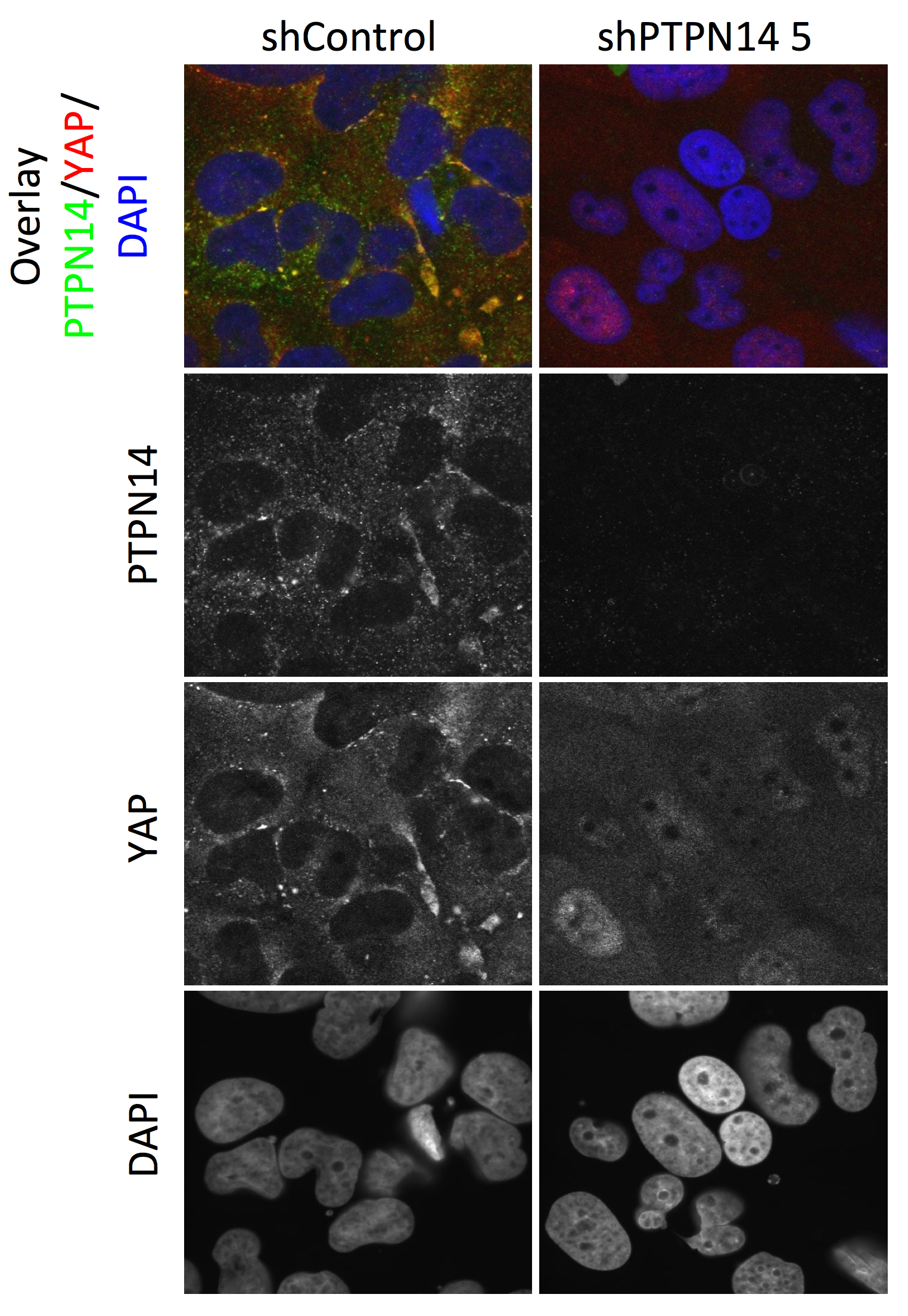

Supplement: Figure S2 — YAP localization in SF268 cells. SF268 cells were transduced with lentivirus encoding for constitutive PTPN14 knock down. Stable cell lines were plated onto Lab-Tek™ 8 chamber glass slides and allowed to reach confluency. Seventy-two hours after seeding, cell lines were analysed by immunofluorescence for PTPN14 and YAP expression using a confocal microscope. DAPI was used for DNA staining. (TIF) [file pone.0061916.s002.tif]
